# Alterations in hepatic miRNA expression during negative energy balance in postpartum dairy cattle

**DOI:** 10.1186/1471-2164-15-28

**Published:** 2014-01-15

**Authors:** Attia Fatima, Sinead Waters, Padraig O’Boyle, Cathal Seoighe, Dermot G Morris

**Affiliations:** 1 School of Mathematics, Statistics and Applied Mathematics National University of Ireland Galway, Galway, Ireland; 2 Animal and Bioscience Research Department, Animal & Grassland Research and Innovation Centre, Teagasc, Mellows Campus, Athenry, Co., Galway, Ireland

## Abstract

**Background:**

Negative energy balance (NEB), an altered metabolic state, occurs in early postpartum dairy cattle when energy demands to support lactation exceed energy intake. During NEB the liver undergoes oxidative stress and increased breakdown of fatty acids accompanied by changes in gene expression. It is now known that micro RNAs (miRNA) can have a role in mediating such alterations in gene expression through repression or degradation of target mRNAs. miRNA expression is known to be altered by metabolism and environmental factors and miRNAs are implicated in expression modulation of metabolism related genes.

**Results:**

miRNA expression was profiled in the liver of moderate yielding dairy cattle under severe NEB (SNEB) and mild NEB (MNEB) using the Affymetrix Gene Chip miRNA_2.0 array with 679 probe sets for *Bos-taurus* miRNAs. Ten miRNAs were found to be differentially expressed using the ‘samr’ statistical package (delta = 0.6) at a q-value FDR of < 12%. Five miRNAs including miR-17-5p, miR-31, miR-140, miR-1281 and miR-2885 were validated using RT-qPCR, to be up-regulated under SNEB. Liver diseases associated with these miRNAs include non-alcoholic fatty liver (NAFLD) and hepatocellular carcinoma (HCC). miR-140 and miR-17-5p are known to show differential expression under oxidative stress. A total of 32 down-regulated putative target genes were also identified among 418 differentially expressed hepatic genes previously reported for the same animal model. Among these, *GPR37* (G protein-coupled receptor 37), *HEYL* (hairy/enhancer-of-split related with YRPW motif-like), *DNJA1*, *CD14* (Cluster of differentiation 14) and *GNS* (glucosamine (N-acetyl)-6-sulfatase) are known to be associated with hepatic metabolic disorders. In addition miR-140 and miR-2885 have binding sites on the most down-regulated of these genes, *FADS2* (Fatty acid desaturase 2) which encodes an enzyme critical in lipid biosynthesis. Furthermore, *HNF3-*gamma (Hepatocyte nuclear factor 3-gamma), a hepatic transcription factor (TF) that is involved in *IGF-1* expression regulation and maintenance of glucose homeostasis is a putative target of miR-31.

**Conclusions:**

This study shows that SNEB affects liver miRNA expression and these miRNAs have putative targets in hepatic genes down-regulated under this condition. This study highlights the potential role of miRNAs in transcription regulation of hepatic gene expression during SNEB in dairy cattle.

## Background

Over the past few decades improvements in milk production through genetic selection have been associated with a reduction in cow fertility and this decrease in fertility has become a major concern for dairy producers [1,2]. Reproduction is an energetically expensive process and an altered metabolic state called NEB has been established as one of the major physiological causes of decreased fertility in high yielding dairy cattle [3-5]. NEB is the result of increased energy demands to support lactation, coupled with lowered feed intake [4,5]. Immune related and hepatic functions are known to be effected by NEB [6-9] and there is an increased metabolic load on the liver to overcome the energy deficit under NEB. There is a dramatic increase in hepatic oxidation of fatty acids for energy production [9,10] and in addition there are extra demands on the liver to increase glycogenesis to meet the glucose requirements of milk production. In order to understand the complex metabolic adjustments in postpartum dairy cattle liver during NEB, a dairy cattle model based on different milking regimes was developed. Earlier in a microarray gene expression study 418 hepatic genes were reported to be differentially expressed as a result of SNEB [9]. These differentially expressed genes have roles in lipid metabolism and glycogenic processes, immune response and the somatotropic axis involved in milk production. The regulation in gene expression under SNEB, however, is yet to be fully understood.

It is now known that a class of small RNAs called miRNA, about 19 to 25 nucleotides in length, can regulate such alterations at the gene expression level. miRNAs were first discovered from the round worm *C. Elegans* almost 3 decades ago [11]. Since their discovery, hundreds of miRNAs have been identified across the plant and animal kingdoms. It is estimated that whereas only 1-5% of genomic transcripts in mammals code miRNAs, up to 60% of genes are regulated by miRNAs [12-14].

The biogenesis of miRNA is a multistep process that begins with miRNA gene transcription resulting in primary miRNA (pri-miRNA) in the nucleus, followed by the generation of around 70nt long stem–loop precursor miRNAs (pre-miRNAs) from pri-miRNA. Pre-miRNAs are then translocated to the cytoplasm where they are trimmed to remove the terminal loop and release ~22 nt long duplex mature miRNAs containing a guiding strand and a passenger strand. The guiding strand assembles into cytoplasmic RNA-induced silencing complex (*RISC)* with argonaute protein that guides the complex to their complementary mRNA targets [15,16]. The miRNA target-mRNA complementary base-pair interaction generally occurs between the target site on the *3′UTR* of the mRNA and the 2^nd^ to 8^th^ nucleotides on the *5′UTR* of the miRNA called the ‘seed region’. Generally, miRNAs repress translation through deadenylation of mRNA leading to their subsequent degradation or translation repression. miRNAs are reported to regulate a wide range of biological processes including cell cycle regulation, proliferation and differentiation as well as development, immune response, carcinogenesis and various metabolic processes [17-19]. The effects of metabolites and environmental factors including hormones, cytokines and nutrients on miRNAs is well established [20-24]. Alterations in cattle liver miRNA expression in response to external anabolic steroids was reported and miRNAs were suggested as potential biomarker for drug abuse in cattle [24]. In another study testosterone treatment was reported to alter miR-22, miR-690, miR-122, let-7a, miR-30 and let-7d expression in female rat liver [25]. Increases in expression of miR-155 and miR-132 due to oxidative stress was reported in an ALD (alchololic liver disorder) mouse model [26]. Oxidative stress of hepatocytes was also reported to alter miR-199a-5p expression [27]. Nutritional modulation of miRNA expression has been reported in various dietary intervention studies of metabolic disorders like obesity, diabetes and fatty liver [28-30]. A high fat diet was reported to alter the adipose tissue miRNA expressional profile of miR-19a, -92a, -92b, -101, -103, -106, -142–5p, and 296 in cattle [31]. miRNAs can regulate metabolism and homeostasis of high energy metabolites as well as insulin signalling and glucose homeostasis [32-34]. miR-33a and miR-33b located within the sterol regulatory element-binding proteins (SREBP), key transcription regulators of genes involved in cholesterol biosynthesis and uptake, regulate cholesterol homeostasis jointly with their host genes [35] and have roles in the regulation fatty acid metabolism and insulin signaling [36]. In addition, miR-122, a liver specific miRNA regulates hepatic fatty acid oxidation and fatty acid and cholesterol synthesis rate [32,37]. miRNAs are associated with the pathophysiology of hepatic metabolic disorders like NAFLD and NASH (non-alcoholic steatohepatitis) as well as HCC in mouse and human studies [38-41]. The potential role miRNAs have to play in NEB in the dairy cow remains to be elucidated.

This study set out to determine a) if hepatic miRNA expression was altered as a result of SNEB in a model of moderate yielding Holstein Friesian dairy cows in the early postpartum period, and b) to integrate hepatic miRNA and mRNA expression profiles through prediction of targets of these miRNAs among a set of previously reported differential expressed hepatic genes under SNEB from the same animal model. Elucidation of the expression patterns of miRNAs and computational identification of their putative target among genes regulated under SNEB will contribute to the understanding of the roles of miRNAs in regulating gene expression during SNEB in dairy cows.

## Methods

### Animal model

The animal model employed in this study has been described previously [5,9]. All procedures were carried out under license in accordance with the European Community Directive, 86-609-EC. In brief, multiparous Holstein-Friesian cows were blocked according to parity, body condition score, and previous milk yield, two weeks prior to expected calving and within block were randomly allocated to two treatments; mild NEB (MNEB, n = 12) or severe NEB (SNEB, n = 12) groups based on different feeding and milking regimes. On day 2 after calving, MNEB cows were fed *ad libitum* grass silage with 8 kg per day of a 21% crude protein dairy concentration and milked once daily; SNEB cows were fed 25 kg of silage per day silage with 4 kg of crude protein per day and milked three times daily. Three times a day versus once a day milking was used to advance a state of SNEB in one group by increasing energy withdrawal with a concomitant lower need for differences in energy intake between groups. The chemical composition of silage and concentrate offered as previously described [5]. For sample collection, cows were selected from each group based on extremes of energy balance (MNEB, n = 6; SNEB, n = 6). Cows were slaughtered approximately 14 days postpartum (MNEB; 13.6 ± 0.75, range 11–15; SNEB 14.3 ± 0.56, range 13–16) [9]. Liver tissues were retrieved within 30 min of slaughter and snap frozen at -80°C. One MNEB cow was removed retrospectively as she was deemed to be ill during the experimental period.

### RNA extraction and quality analysis

For the purpose of RNA extraction, 100 mg frozen liver tissue was directly immersed in 1 ml Trizol (Invitrogen, Stockholm, Sweden). A Precellys 24 homogeniser (Bertin Technologies, Montigny-le-Bretonneux, France) was used to homogenise the tissue (6000 rpm for 20 sec). Homogenate was left for 5 min at RT to permit the complete dissociation of nucleoprotein complexes followed by centrifugation at 12,000 g for 10 min at 4°C. Supernatant was transferred to a 2 ml tube. 2μl of Pellet paint® NF Co-Precipitant (EMD Millipore, Darmstadt, Germany) and 200 μl chloroform was added and incubated at RT for 5 min. After centrifugation for 15 min at 4°C and 14000 g the aqueous phase was transferred into a new tube and 500 μl isopropanol was added and incubated for 5 min on ice. Following centrifugation for 10 min at 4°C and 14000 g the supernatant was removed and the pellet washed with 500 μl 75% ETOH followed by centrifugation for 10 min at 4°C and 14000 g. A 100 μl aliquot of nuclease-free water was added to the pellet and incubated at RT for 10 min. After vortexing for 1 min 10 μl of 3 M sodium acetate was added. Next, 250 μl ice cold ETOH was added and incubated for 1 hr at -20°C, followed by centrifugation at 4°C for 10 min and 14000 g. The supernatant was removed and washed with 500 μl of 75% EtOH by vortexing followed by centrifugation at 4°C for 10 min and 14000 g. The supernatant was removed and the pellet was air-dried for 5 min at RT following which 30μl of RNase-free water was added and left for 5 min. RNA quality and quantity was assessed using a NanoDrop spectrophotometer (ND-1000; Wilmington, DE, USA) and Bioanalyzer 2100 (Agilent Technologies Ireland, Dublin, Ireland). Mean RIN values were > 8. Total RNA was stored at -80°C.

### Gene Chip miRNAs_2.0 array hybridization

Total RNA from liver samples of 11 cows were hybridized to the Affymetrix Gene Chip miRNA_2.0 (Affymetrix, Santa Clara, CA, USA) arrays for miRNA expression profiling. This array has miRBase v15 coverage (www.mirbase.org) with 15,644 probe sets of 131 organisms including 679 probe sets for *Bos*-*taurus* miRNAs. Array hybridization was carried out at ATLAS Biolabs, Berlin, Germany. Briefly, 200 ng total RNA labelled with Flash Tag Biotin was hybridized to the miRNA Array in a Hybridization Oven 640 (Affymetrix) at 48°C for 18 h. The arrays were stained with the Fluidics Station 450 using fluidics script FS450_0003 (Affymetrix), and then scanned on a GeneChip Scanner 3000 7G (Affymetrix, Santa Clara, CA, USA).

### Statistical analysis

All statistical analyses were performed in the R statistical computing environment (Version 2.14; http://www.r-project.org) with the *samr* package from the Bioconductor project (http://www.bioconductor.org). Data quality was assessed with the *ArrayQualitymetrix* package from Bioconductor [42,43]. One cel file from each group was discarded due to issues with hybridisation quality. The *expresso* method of the Affy package [44] was used to pre-processes the data with quantile normalization and median polish summarization. While the chip contains probes complementary to miRNA from other species only the expression intensities of 679 cattle miRNAs were used for further analysis. The Significance Analysis of Microarray (SAM) two-class unpaired method implemented in the *samr* Bioconductor package was used to identify differentially expressed miRNAs [45]. The *samr* parameter delta 0.6 was selected that classified miRNAs as differentially expressed at a fold-change > 1.25 and a q-value < 12%.

### RT-qPCR validation of differentially expressed miRNAs

Seven candidate miRNAs differentially expressed on the microarray; including miR-17-5p, miR-31, miR-140, miR-1281, miR-2885, miR-296, and miR-671 were selected for RT-qPCR validation. miRNA expression was carried out with TaqMan miRNA RT-qPCR assays according to the manufacturer’s instructions (Applied Biosystems, Dublin, Ireland). miRNA-specific reverse transcription was performed on 10 ng of purified total RNA using the TaqMan MicroRNA Reverse Transcription kit according to manufacturer’s instructions. RT-qPCR reactions were performed using 1 μl of cDNA (10 ng/μl) in 9 μl of Taqman universal master mix containing TaqMan PCR primers and probes on a BioRad CFX96 real time PCR system (Bio-Rad, Hemel Hempstead, UK) using the following cycling parameters; 95°C for 10 min followed by 40 cycles at 95°C for 15 s and 60°C for 1 min. Four biological replicates from each of the MNEB and SNEB treatment groups were used for RT-qPCR validation. Three reference miRNAs including miR-122, let-7b and *RNU6B* were tested with the software program geNorm version 3.5 [46] for calculating the gene expression stability measure (M value). *RNU6B* was found to be the most stable internal reference miRNA across treatments with an M value of 0.7. It was more stable on its own than when used in combination with the two other reference miRNA. The software package BioRad CFX manager was used for correction of the Ct values and normalization to RNU6B using the 2^-ΔΔCt^ method [47]. Corrected Ct values were used to calculate differential expression using the PROC t-test (SAS) [48].

### Prediction of differentially expressed miRNAs targets among differentially expressed hepatic genes under SNEB

A target prediction algorithm for custom data, from the TargetScan database website [13] implemented in Perl (targetscan_61_context_scores.pl), was used to identify target sites of differentially expressed miRNAs in the 3′UTRs of 418 differentially expressed genes reported previously for the same liver tissue [9]. Using this algorithm, conserved 8mer and 7mer sites matching the seed region of each miRNA in the 3′UTRs of differentially expressed hepatic genes were identified [49]. Non-conserved sites and sites with mismatches in the seed region that were compensated by conserved 3′ pairing were also included [13]. Predictions were ranked based on the *context + scores* of the sites that represent the predicted efficacy of targeting [50]. GO (Gene Ontology) biological processes associations of 3 conserved miRNAs (miR-31, miR 17-5p and miR-140) were retrieved using FAME (functional assignment of miRNAs via enrichment) software [51]. No data was available for miR-1281 and miR-2885 in the FAME database. The FAME algorithm makes direct inference of a specific miRNA function using enriched subsets of the target genes sharing a common biological process or pathway. The GO biological processes associated with down-regulated putative targets were retrieved from the UniProt Gene Ontology Annotation (GOA) database (http://www.ebi.ac.uk/GOA).

## Results

Ten out of 679 mature bovine miRNAs represented on the miRNA Gene Chip miRNA_2.0 array were found to be up-regulated in SNEB cows. Table 1 lists the miRNAs that showed differential expression between MNEB and SNEB groups. RT-qPCR results confirmed the differential expression of 5 out of the 7 miRNAs tested. The expression of miR17-5p, -1281, -140, 2885 and -31 were consistent between microarray and RT-qPCR whereas miR-296 and miR-671 were not significant. Table 2 lists the differentially expressed miRNAs validated by RT-qPCR. Putative targets of these five miRNAs were identified in the set of 418 (230 down- and 188 up-regulated) hepatic genes reported previously in the same animal model. Among the 418, 67 (35 up-regulated and 32 down-regulated) genes were found to have target sites in the 3′UTRs of the up-regulated miRNAs (Table 3).

**Table 1:** Differentially expressed post-partum dairy cattle miRNAs under SNEB

**miRNA**	**miRBase accession**	**q-value (%)**	**Fold change**
bta-miR-31	MI0004762	0.00	1.79
bta-miR-1281	MI0010466	11.97	1.71
bta-miR-2483	MI0011545	11.97	1.56
bta-miR-2885	MI0013058	0.00	1.55
bta-miR-296	MI0009786	11.97	1.52
bta-miR-2316	MI0011336	11.97	1.40
bta-miR-140	MI0005010	11.97	1.33
bta-miR-17-5p	MIMAT0003815	11.97	1.30
bta-miR-671	MI0009887	11.97	1.29
bta-miR-2455	MI0011512	11.97	1.28

Differential expression analysis was done for microarray expression intensities of 679 bovine miRNAs with SAM (significance analysis of microarrays) method at delta parameter of 0.6 and q FDR < 12%.

**Table 2:** Differentially expressed postpartum dairy cattle hepatic miRNAs under SNEB

**miRNA**	**Fold change**	**p value**
bta-miR-31	4.11	0.033
bta-miR-1281	3.10	0.042
bta-miR-2885	2.91	0.006
bta-miR-17-5p	4.62	0.008
bta-miR-140	3.90	0.030
bta-miR-296	0.85	0.490
bta-miR-671	0.92	0.930

Differential expression analysis was carried out with PROC t-test (SAS) after normalization of Ct-values to reference gene RNU6B.

**Table 3:** Putative target genes of up-regulated hepatic miRNAs differentially expressed under SNEB

**Down-regulated putative targets**		**Up-regulated putative targets**	
**Symbol**	**Gene name**	**Symbol**	**Gene name**
*ALDH1A1*	aldehyde dehydrogenase 1 family, member A1	*AADAT*	aminoadipate aminotransferase
*BTG1*	B-cell translocation gene 1, anti-proliferative	*ACP2*	Acid phosphatase 2, lysosomal
*CCL19*	chemokine (C-C motif) ligand 19	*ADTRP*	Androgen-Dependent TFPI-Regulating Protein
*CD14*	CD14 molecule	*ARG1*	Arginase, liver
*CIB1*	Calcium and integrin binding 1 (calmyrin)	*BIN1*	Bridging integrator 1
*DNAJA1*	DnaJ (Hsp40) homolog, subfamily A, member 1	*BNIP3L*	BCL2/adenovirus E1B 19 kDa interacting protein 3-like
*DSG1*	desmoglein 1	*CMPK1*	cytidine monophosphate (UMP-CMP) kinase 1, cytosolic
*EPB41L5*	Erythrocyte membrane protein band 4.1 like 5	*CNN1*	Calponin 1, Basic, Smooth Muscle
*ERRFI1*	ERBB receptor feedback inhibitor 1	*CPQ*	carboxypeptidase Q
*FADS2*	Fatty acid desaturase 2	*CPT1B*	carnitine palmitoyltransferase 1B (muscle)
*FOXA3*	Forkhead box A3	*DENND2D*	DENN/MADD domain containing 2D
*FXR1*	Fragile X mental retardation, autosomal homolog 1	*DPYD*	dihydropyrimidine dehydrogenase
*GKAP1*	G kinase anchoring protein 1	*HDC*	histidine decarboxylase
*GNS*	glucosamine (N-acetyl)-6-sulfatase	*HMGN4*	High mobility group nucleosomal binding domain 4
*GPBP1*	GC-rich promoter binding protein 1	*HSF2BP*	Heat shock transcription factor 2 binding protein
*GPR37*	G protein-coupled receptor 37 (endothelin receptor type B-like)	*IL1A*	Interleukin 1, alpha
*HEYL*	hairy/enhancer-of-split related with YRPW motif-like	*LPCAT3*	Lysophosphatidylcholine acyltransferase 3
*IP6K2*	inositol hexakisphosphate kinase 2	*MBL2*	Mannose-binding lectin (protein C) 2, soluble (opsonic defect)
*KIAA1 191*	KIAA1 191	*MINA*	MYC induced nuclear antigen
*MIEN1*	Migration and invasion enhancer 1Bottom of Form	*NHEJ1*	Nonhomologous end-joining factor 1
*MIS 12*	MIND kinetochore complex component, homolog (S. pombe)	*OAT*	ornithine aminotransferase (gyrate atrophy)
*NR2F1*	nuclear receptor subfamily 2, group F, member 1	*PDRG1*	p53 and DNA-damage regulated 1
*PRSS35*	protease, serine, 35	*POLR3GL*	polymerase (RNA) III polypeptide G (32kD)-like
*PTPRR*	protein tyrosine phosphatase, receptor type, R	*RCAN3*	RCAN family member 3
*RNF 128*	ring finger protein 128	*REEP5*	Receptor accessory protein 5

Putative binding sites were identified in the 3′UTR of differentially expressed hepatic genes under SNEB with the Targetscan algorithm.

**Figure 1:**
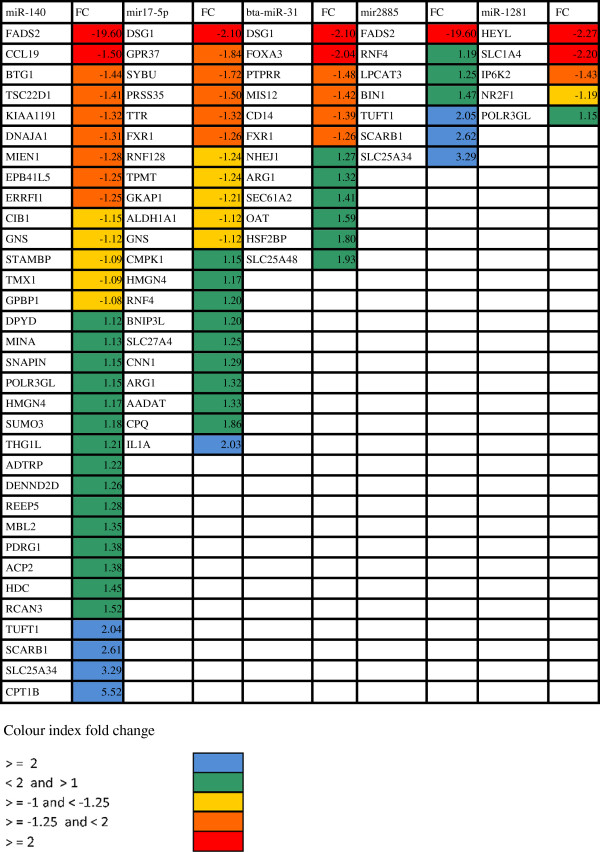
Predicted miRNA gene targets with fold-change under SNEB.

**Figure 2:**
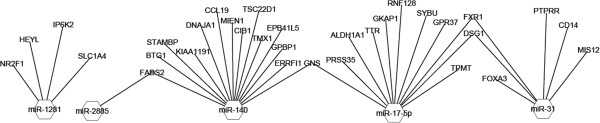
**Hexagons represent the up-regulated miRNAs with edges leading to nodes representing their putative targets down-regulated under SNEB.** Nodes with two edges represent putative targets shared by two up-regulated miRNAs.

All of the five differentially expressed miRNAs validated by RT-qPCR in this study were up-regulated however there was no significant enrichment among the down-regulated putative targets genes using the Fisher test for enrichment analysis. Overall miR-140 was found to have the most putative targets among differentially expressed genes under SNEB while miR-17-5p had the second highest number of targets. As the consensus is that up-regulated miRNAs result for the most part in down-regulation of their target genes, we further selected only down-regulated genes as putative affected targets for subsequent analysis to investigate the role of differentially expressed miRNAs under SNEB. As miRNAs are thought not to switch off their targets completely but rather fine-tune their expression, all down-regulated genes were included irrespective of fold-change. Figure 1 presents the subset of the putative down-regulated targets for these up-regulated miRNAs. miR-140 had the highest number of putative down-regulated target genes followed by miR-17-5p. miR-31 was the most up-regulated miRNA under SNEB while miR-1281 had all but one of its putative target genes down-regulated under SNEB. miR-2885 has *FADS2* as its only putative down-regulated target under SNEB and *FADS2* is also a putative target of miR-140. *DSG1* (desmoglein1) and *FXR1* (fragile X mental retardation, autosomal homolog 1) are both shared targets of miR-17-5p and miR-31 while *GNS* (glucosamine-N-acetyl-6-sulfatase) is a shared target of mir-140 and miR-31 (Figure 2). Table 4 lists the bio-types of putative targets down-regulated under SNEB. The GO biological processes associated with down-regulated putative targets are given in Additional file 1: Table S1. The overall GO biological functions associated with three conserved miRNAs that are relevant in terms of SNEB are given in Additional file 1: Table S2. miR-31 is associated with the GO biological processes of cell growth, amino acid transport and response to stress. miR-17-5p is associated with GO biological processes include regulation of cell cycle and protein metabolism. miR-140 is associated with GO biological processes of cell proliferation, polysaccharide metabolism and signal transduction, while both miR-31 and miR-140 are associated with response to nutritional level.

**Table 4:** Intracellular location and bio-types of down-regulated putative gene targets of up-regulated miRNAs

**Symbol**	**Entrez gene name**	**Location**	**Type**
KIAA1191	KIAA1191	Cytoplasm	Other
MIEN 1	Migration and invasion enhancer 1	Cytoplasm	Other
MIS 12	MIS 12, MIND kinetochore complex component, homolog (S. pombe)	Nucleus	Other
NR2F1	Nuclear receptor subfamily 2, group F, member 1	Nucleus	Ligand-dependent nuclear receptor
PRSS35	Protease, serine, 35	Extracellular space	peptidase
PTPRR	Protein tyrosine phosphatase, receptor type, R	Plasma membrane	phosphatase
RNF 128	Ring finger protein 128, E3 ubiquitin protein ligase	Cytoplasm	Enzyme
SLC1A4	Solute carrier family 1 (glutamate/neutral amino acid transporter), member 4	Plasma membrane	Transporter
STAMBP	STAM binding protein	Nucleus	Enzyme
SYBU	Syntabulin (syntaxin-interacting)	Unknown	Other
TMX1	Thioredoxin-related transmembrane protein 1	Cytoplasm	Enzyme
TPMT	Thiopurine S-methyltransferase	Cytoplasm	Enzyme
TTR	Transthyretin1	Cytoplasm	Transporter
ALDH1A1	Aldehyde dehydrogenase 1 family, member A1	Cytoplasm	Enzyme
BTG1	B-cell Translocation gene 1, anti-proliferative	Nucleus	Transcription Regulator
CCL19	chemokine (C-C motif) ligand 19	Extracellular space	Cytokine
CD14	CD14 molecule	Plasma Membrane	Transmembrane receptor
CIB1	Calcium and integrin binding 1 (calmyrin)	Nucleus	Other
DNAJA1	DnaJ (Hsp40) homolog, subfamily A, member 1	Nucleus	Other
DSG1	Desmoglein 1	Plasma membrane	Membrane protein
EPB4 1 L5	Erythrocyte membrane protein band 4.1 like 5	Plasma membrane	Membrane protein
ERRFI1	ERBB receptor feedback inhibitor 1	Cytoplasm	Other
FADS2	Fatty acid desaturase 2	Plasma membrane	Enzyme
FOXA3	Forkhead box A3	Nucleus	Transcription regulator
FXR1	Fragile X mental retardation, autosomal homolog 1	Cytoplasm	Other
GKAP1	G kinase anchoring protein 1	Cytoplasm	Other
GNS	Glucosamine (N-acetyl)-6-sulfatase	Cytoplasm	Enzyme
GPBP1	GC-rich promoter binding protein 1	Nucleus	Transcription regulator
GPR37	G protein-coupled receptor 37 (endothelin receptor type B-like)	Plasma membrane	G-protein coupled receptor
HEYL	Hairy/enhancer-of-split related with YRPW motif-like	Nucleus	Transcription regulator
IP6K2	Inositol hexakisphosphate kinase 2	Cytoplasm	Kinase

## Discussion

Dairy cows enter a state of NEB postpartum when the energy supply is prioritised towards the mammary tissues for milk production while the energy intake is reduced. The principal physiological response to the energy deficit is mobilization of non-esterified fatty acids (NEFA) from adipose tissue. NEFAs are broken down in the liver to release energy and any incomplete breakdown results in production of *BHB* (β-hydroxybutyrate) or *TAGs* (triacylglycerides). Accumulation of *TAGs, BHB* and NEFAs results in the onset of oxidative stress in the liver that can lead to fatty liver or lipidosis. In addition components of the somatotrophic axis like *IGF* (insulin like growth factor) and *GH* (growth hormones) that have roles in the control of milk production are also affected by SNEB [8-10]. Reduced *IGF1* expression is also negatively correlated with fertility in dairy cows [52]. Previous nutritional supplementation of animals in NEB with lipogenic and glycogenic nutrients partly succeeded in compensating for energy partitioning between mammary tissues and the rest of the body [4,53]. However, the regulation of such alterations is yet to be explored. In this study, five miRNAs were altered in response to SNEB. Two of these miRNAs have been reported to be altered under oxidative stress in humans [41,54] and miR-17-5p has been associated with oxidative stress in HCC [41]. In addition alterations in miR-140 expression under chemical and oxidative stress has also been reported [54]. Three of the miRNAs, miR- 17, miR-31 and miR-140, found to be up-regulated under SNEB have been previously associated with hepatic disorders [55-58] and miR-140 has been associated with NAFLD [40,57]. A regulatory role for miR-17-5p has been reported in HCC [55,56] while miR-31 has also been associated with both NAFLD and HCC [58-60].

A number of the putative down-regulated targets of the differentially expressed miRNAs in this study are known to have roles in hepatic disorders. These include the miR-17-5p target *GPR37* (G protein-coupled receptor 37) a member of the G-protein coupled receptor-1 family associated with NAFLD [61]. The miR-1281 target *HEYL* (hairy/enhancer-of-split related with YRPW motif-like) is associated with HCC [62,63]. The miR-140 target *DNJA1* that has a role in lipid intake is associated with the oxidative stress response of high fat induced NAFLD [28,30]. The miR-31 target, *CD14* (Cluster of differentiation 14) involved in transmission and release of polysaccharides, is also implicated in ALD, hepatic cholestasis and hepatic fibrosis [64,65]. The miR-17-5p target *GNS* (glucosamine (N-acetyl)-6-sulfatase) that metabolises glucosamine sulphate has been implicated in hepatocellular dysplasia, cirrhosis of the liver and hepatic fibrosis [66]. Moreover, the miR-17-5p target *TTR,* a carrier protein for transport of lipid soluble vitamins from the liver to the circulation is also reduced under SNEB. Reduced *TTR* expression has been reported in a study of periparturient period Holstein and Jersey dairy cows in NEB [67]. In addition we were able to postulate a role for miR-31 in SNEB through its putative target hepatic transcription factor *FOXA3* also known as *HNF3-gamma.* Bovine *IGF-I* has binding sites for *HNF3-gamma* in its promoter region [68]. While another recent human study reported target sites of miR-31 on the 3′UTR of *IGF-1*[69]. *HNF-*gamma is also known to be involved in glucose homeostasis in hepatocytes through regulation of *GLUT2* (glucose transporter 2) [70,71]. However, the most interesting putative target of all was the gene encoding the lipogenesis enzyme *FADS2* which is critical for long-chain polyunsaturated fatty acids biosynthesis [72]. In this study we found binding sites for two up-regulated miRNAs miR-140 and miR-2885 in the 3′UTR of *FADS2,* whereas miR-140 has been previously implicated in the regulation of *FADS2* homolog *FADS1*[73].

## Conclusion

This study suggests a role for hepatic miRNAs in lipid and energy metabolism through the identification of a subset of their putative targets associated with important metabolic processes. Moreover hepatic miRNAs associated with genes involved in the somatotropic axis can be a possible link between reduced reproductive performance and SNEB in the early postpartum dairy cow. Further direct functional studies of selected miRNA-mRNA putative pairs in the liver of such models will help to improve our understanding of the molecular mechanisms and pathways involved in SNEB in postpartum dairy cattle.

### Availability of supporting data

The data sets supporting the results of this article are available in Gene Expression Omnibus (GEO) repository (GSE51658).

## Competing interests

The authors declare that they have no competing interests.

## Authors’ contributions

DM, CS and SW conceived and designed the experiments. AF and PO performed the experiments. AF, CS and DM analyzed the data. AF, DM and CS drafted the manuscript. All authors read and approved the final manuscript.

## Authors’ information

Cathal Seoighe and Dermot G Morris are joint senior authors.

## Supplementary Material

Additional file 1: Table S1 GO biological processes associated with putative target genes down-regulated under SNEB. **Table S2.** FAME GO biological processes associated with up-regulated miRNAs under SNEB. Click here for file
