# Supplementary material for: Alterations in hepatic miRNA expression during negative energy balance in postpartum dairy cattle
Source: BMC Genomics. 2014 Jan 15;15:28. doi: 10.1186/1471-2164-15-28 (PMC3902422; doi:10.1186/1471-2164-15-28)
Supplement: Additional file 1: Table S1 — GO biological processes associated with putative target genes down-regulated under SNEB. Table S2. FAME GO biological processes associated with up-regulated miRNAs under SNEB. [file 1471-2164-15-28-S1.doc]

**Supplementary Table 1.** GO biological processes associated with putative target genes down-regulated under SNEB

| **Symbol** | **ENTREZ GENE Name** | **GO Biological Process** |
| --- | --- | --- |
| *TTR* | Transthyretin1 |  |
|  |  | extracellular matrix organization |
|  |  | [phototransduction, visible light](http://amigo.geneontology.org/cgi-bin/amigo/go.cgi?view=details&depth=1&query=7603) |
|  |  | [retinoid metabolic process](http://amigo.geneontology.org/cgi-bin/amigo/go.cgi?view=details&depth=1&query=1523) |
|  |  | [retinol metabolic process](http://amigo.geneontology.org/cgi-bin/amigo/go.cgi?view=details&depth=1&query=42572) |
|  |  | [transport](http://amigo.geneontology.org/cgi-bin/amigo/go.cgi?view=details&depth=1&query=6810) |
| *TPMT* | Thiopurine S-methyltransferase |  |
|  |  | [methylation](http://amigo.geneontology.org/cgi-bin/amigo/go.cgi?view=details&depth=1&query=32259) |
|  |  | nucleobase-containing compound metabolic process |
|  |  | [small molecule metabolic process](http://amigo.geneontology.org/cgi-bin/amigo/go.cgi?view=details&depth=1&query=44281) |
|  |  | [xenobiotic metabolic process](http://amigo.geneontology.org/cgi-bin/amigo/go.cgi?view=details&depth=1&query=6805) |
| *TMX1* | Thioredoxin-related transmembrane protein 1 |  |
|  |  | [DNA replication](http://amigo.geneontology.org/cgi-bin/amigo/go.cgi?view=details&depth=1&query=6260) |
|  |  | [ER to Golgi vesicle-mediated transport](http://amigo.geneontology.org/cgi-bin/amigo/go.cgi?view=details&depth=1&query=6888) |
|  |  | [cell proliferation](http://amigo.geneontology.org/cgi-bin/amigo/go.cgi?view=details&depth=1&query=8283) |
|  |  | [cell redox homeostasis](http://amigo.geneontology.org/cgi-bin/amigo/go.cgi?view=details&depth=1&query=45454) |
|  |  | [leukocyte activation](http://amigo.geneontology.org/cgi-bin/amigo/go.cgi?view=details&depth=1&query=45321) |
|  |  | [negative regulation of apoptotic process](http://amigo.geneontology.org/cgi-bin/amigo/go.cgi?view=details&depth=1&query=43066) |
|  |  | [positive regulation of growth](http://amigo.geneontology.org/cgi-bin/amigo/go.cgi?view=details&depth=1&query=45927) |
|  |  | [positive regulation of transcription, DNA-dependent](http://amigo.geneontology.org/cgi-bin/amigo/go.cgi?view=details&depth=1&query=45893) |
|  |  | [response to stress](http://amigo.geneontology.org/cgi-bin/amigo/go.cgi?view=details&depth=1&query=6950) |
|  |  | [signal transduction](http://amigo.geneontology.org/cgi-bin/amigo/go.cgi?view=details&depth=1&query=7165) |
| *STAMBP* | STAM binding protein |  |
|  |  | [JAK-STAT cascade](http://amigo.geneontology.org/cgi-bin/amigo/go.cgi?view=details&depth=1&query=7259) |
|  |  | [mitotic cytokinesis](http://amigo.geneontology.org/cgi-bin/amigo/go.cgi?view=details&depth=1&query=281) |
|  |  | [negative regulation of Ras protein signal transduction](http://amigo.geneontology.org/cgi-bin/amigo/go.cgi?view=details&depth=1&query=46580) |
|  |  | [negative regulation of apoptotic process](http://amigo.geneontology.org/cgi-bin/amigo/go.cgi?view=details&depth=1&query=43066) |
|  |  | [negative regulation of phosphatidylinositol 3-kinase cascade](http://amigo.geneontology.org/cgi-bin/amigo/go.cgi?view=details&depth=1&query=14067) |
|  |  | [positive regulation of cell proliferation](http://amigo.geneontology.org/cgi-bin/amigo/go.cgi?view=details&depth=1&query=8284) |
|  |  | [protein deubiquitination](http://amigo.geneontology.org/cgi-bin/amigo/go.cgi?view=details&depth=1&query=16579) |
|  |  | [proteolysis](http://amigo.geneontology.org/cgi-bin/amigo/go.cgi?view=details&depth=1&query=6508) |
| *SLC1A4* | Solute carrier family 1 (glutamate/neutral amino acid transporter), member 4 |  |
|  |  | [L-alanine transport](http://amigo.geneontology.org/cgi-bin/amigo/go.cgi?view=details&depth=1&query=15808) |
|  |  | [L-cystine transport](http://amigo.geneontology.org/cgi-bin/amigo/go.cgi?view=details&depth=1&query=15811) |
|  |  | [NOT L-cystine transport](http://amigo.geneontology.org/cgi-bin/amigo/go.cgi?view=details&depth=1&query=15811) |
|  |  | [L-serine transport](http://amigo.geneontology.org/cgi-bin/amigo/go.cgi?view=details&depth=1&query=15825) |
|  |  | [amino acid transport](http://amigo.geneontology.org/cgi-bin/amigo/go.cgi?view=details&depth=1&query=6865) |
|  |  | [chloride transport](http://amigo.geneontology.org/cgi-bin/amigo/go.cgi?view=details&depth=1&query=6821) |
|  |  | [cognition](http://amigo.geneontology.org/cgi-bin/amigo/go.cgi?view=details&depth=1&query=50890) |
|  |  | [glutamine transport](http://amigo.geneontology.org/cgi-bin/amigo/go.cgi?view=details&depth=1&query=6868) |
|  |  | [hydroxyproline transport](http://amigo.geneontology.org/cgi-bin/amigo/go.cgi?view=details&depth=1&query=34589) |
|  |  | [ion transport](http://amigo.geneontology.org/cgi-bin/amigo/go.cgi?view=details&depth=1&query=6811) |
|  |  | [proline transmembrane transport](http://amigo.geneontology.org/cgi-bin/amigo/go.cgi?view=details&depth=1&query=35524) |
|  |  | [proline transport](http://amigo.geneontology.org/cgi-bin/amigo/go.cgi?view=details&depth=1&query=15824) |
|  |  | [synaptic transmission, glutamatergic](http://amigo.geneontology.org/cgi-bin/amigo/go.cgi?view=details&depth=1&query=35249) |
|  |  | [threonine transport](http://amigo.geneontology.org/cgi-bin/amigo/go.cgi?view=details&depth=1&query=15826) |
|  |  | [transmembrane transport](http://amigo.geneontology.org/cgi-bin/amigo/go.cgi?view=details&depth=1&query=55085) |
| *RFP128* | Ring finger protein 128, E3 ubiquitin protein ligase |  |
|  |  | [negative regulation of cytokine biosynthetic process](http://amigo.geneontology.org/cgi-bin/amigo/go.cgi?view=details&depth=1&query=42036) |
| *PTPRR* | Protein tyrosine phosphatase, receptor type, R |  |
|  |  | [in utero embryonic development](http://amigo.geneontology.org/cgi-bin/amigo/go.cgi?view=details&depth=1&query=1701) |
|  |  | [protein dephosphorylation](http://amigo.geneontology.org/cgi-bin/amigo/go.cgi?view=details&depth=1&query=6470) |
| *NR2F1* | Nuclear receptor subfamily 2, group F, member 1 |  |
|  |  | [gene expression](http://amigo.geneontology.org/cgi-bin/amigo/go.cgi?view=details&depth=1&query=10467) |
|  |  | [intracellular receptor signaling pathway](http://amigo.geneontology.org/cgi-bin/amigo/go.cgi?view=details&depth=1&query=30522) |
|  |  | [negative regulation of transcription from RNA polymerase II promoter](http://amigo.geneontology.org/cgi-bin/amigo/go.cgi?view=details&depth=1&query=122) |
|  |  | [neuron migration](http://amigo.geneontology.org/cgi-bin/amigo/go.cgi?view=details&depth=1&query=1764) |
|  |  | [signal transduction](http://amigo.geneontology.org/cgi-bin/amigo/go.cgi?view=details&depth=1&query=7165) |
|  |  | [transcription initiation from RNA polymerase II promoter](http://amigo.geneontology.org/cgi-bin/amigo/go.cgi?view=details&depth=1&query=6367) |
| *MIS12* | MIS 12, MIND kinetochore complex component, homolog (S. pombe |  |
|  |  | [cell division](http://amigo.geneontology.org/cgi-bin/amigo/go.cgi?view=details&depth=1&query=51301) |
|  |  | [chromosome segregation](http://amigo.geneontology.org/cgi-bin/amigo/go.cgi?view=details&depth=1&query=7059) |
|  |  | [kinetochore assembly](http://amigo.geneontology.org/cgi-bin/amigo/go.cgi?view=details&depth=1&query=51382) |
|  |  | [mitosis](http://amigo.geneontology.org/cgi-bin/amigo/go.cgi?view=details&depth=1&query=7067) |
|  |  | [mitotic cell cycle](http://amigo.geneontology.org/cgi-bin/amigo/go.cgi?view=details&depth=1&query=278) |
| *MIEN 1* | Migration and invasion enhancer 1 |  |
|  |  | [apoptotic process](http://amigo.geneontology.org/cgi-bin/amigo/go.cgi?view=details&depth=1&query=6915) |
|  |  | [cell redox homeostasis](http://amigo.geneontology.org/cgi-bin/amigo/go.cgi?view=details&depth=1&query=45454) |
|  |  | [negative regulation of apoptotic process](http://amigo.geneontology.org/cgi-bin/amigo/go.cgi?view=details&depth=1&query=43066) |
|  |  | [positive regulation of cell migration](http://amigo.geneontology.org/cgi-bin/amigo/go.cgi?view=details&depth=1&query=30335) |
|  |  | [positive regulation of filopodium assembly](http://amigo.geneontology.org/cgi-bin/amigo/go.cgi?view=details&depth=1&query=51491) |
| *BTG1* | B-cell translocation gene 1, anti-proliferative |  |
|  |  | [cell migration](http://amigo.geneontology.org/cgi-bin/amigo/go.cgi?view=details&depth=1&query=16477) |
|  |  | [negative regulation of cell growth](http://amigo.geneontology.org/cgi-bin/amigo/go.cgi?view=details&depth=1&query=30308) |
|  |  | [negative regulation of cell proliferation](http://amigo.geneontology.org/cgi-bin/amigo/go.cgi?view=details&depth=1&query=8285) |
|  |  | [positive regulation of angiogenesis](http://amigo.geneontology.org/cgi-bin/amigo/go.cgi?view=details&depth=1&query=45766) |
|  |  | [positive regulation of endothelial cell differentiation](http://amigo.geneontology.org/cgi-bin/amigo/go.cgi?view=details&depth=1&query=45603) |
|  |  | [positive regulation of fibroblast apoptotic process](http://amigo.geneontology.org/cgi-bin/amigo/go.cgi?view=details&depth=1&query=2000271) |
|  |  | [positive regulation of myoblast differentiation](http://amigo.geneontology.org/cgi-bin/amigo/go.cgi?view=details&depth=1&query=45663) |
|  |  | [regulation of transcription, DNA-dependent](http://amigo.geneontology.org/cgi-bin/amigo/go.cgi?view=details&depth=1&query=6355) |
| *ALDH1A1* | Aldehyde dehydrogenase 1 family, member A1 |  |
|  |  | [cellular aldehyde metabolic process](http://amigo.geneontology.org/cgi-bin/amigo/go.cgi?view=details&depth=1&query=6081) |
|  |  | [ethanol oxidation](http://amigo.geneontology.org/cgi-bin/amigo/go.cgi?view=details&depth=1&query=6069) |
|  |  | [positive regulation of Ras GTPase activity](http://amigo.geneontology.org/cgi-bin/amigo/go.cgi?view=details&depth=1&query=32320) |
|  |  | [retinol metabolic process](http://amigo.geneontology.org/cgi-bin/amigo/go.cgi?view=details&depth=1&query=42572) |
|  |  | [small molecule metabolic process](http://amigo.geneontology.org/cgi-bin/amigo/go.cgi?view=details&depth=1&query=44281) |
|  |  | [xenobiotic metabolic process](http://amigo.geneontology.org/cgi-bin/amigo/go.cgi?view=details&depth=1&query=6805) |
| *CL19* | Chemokine (C-C motif) ligand 19 |  |
|  |  | [T cell costimulation](http://amigo.geneontology.org/cgi-bin/amigo/go.cgi?view=details&depth=1&query=31295) |
|  |  | [activation of JUN kinase activity](http://amigo.geneontology.org/cgi-bin/amigo/go.cgi?view=details&depth=1&query=7257) |
|  |  | [cell communication](http://amigo.geneontology.org/cgi-bin/amigo/go.cgi?view=details&depth=1&query=7154) |
|  |  | [cell maturation](http://amigo.geneontology.org/cgi-bin/amigo/go.cgi?view=details&depth=1&query=48469) |
|  |  | [cellular calcium ion homeostasis](http://amigo.geneontology.org/cgi-bin/amigo/go.cgi?view=details&depth=1&query=6874) |
|  |  | [dendritic cell chemotaxis](http://amigo.geneontology.org/cgi-bin/amigo/go.cgi?view=details&depth=1&query=2407) |
|  |  | [establishment of T cell polarity](http://amigo.geneontology.org/cgi-bin/amigo/go.cgi?view=details&depth=1&query=1768) |
|  |  | [immune response](http://amigo.geneontology.org/cgi-bin/amigo/go.cgi?view=details&depth=1&query=6955) |
|  |  | [immunological synapse formation](http://amigo.geneontology.org/cgi-bin/amigo/go.cgi?view=details&depth=1&query=1771) |
|  |  | [inflammatory response](http://amigo.geneontology.org/cgi-bin/amigo/go.cgi?view=details&depth=1&query=6954) |
|  |  | [interleukin-12 secretion](http://amigo.geneontology.org/cgi-bin/amigo/go.cgi?view=details&depth=1&query=72610) |
|  |  | [mature dendritic cell differentiation](http://amigo.geneontology.org/cgi-bin/amigo/go.cgi?view=details&depth=1&query=97029) |
|  |  | [myeloid dendritic cell chemotaxis](http://amigo.geneontology.org/cgi-bin/amigo/go.cgi?view=details&depth=1&query=2408) |
|  |  | [negative regulation of leukocyte apoptotic process](http://amigo.geneontology.org/cgi-bin/amigo/go.cgi?view=details&depth=1&query=2000107) |
|  |  | [positive regulation of Cdc42 GTPase activity](http://amigo.geneontology.org/cgi-bin/amigo/go.cgi?view=details&depth=1&query=43089) |
|  |  | [positive regulation of ERK1 and ERK2 cascade](http://amigo.geneontology.org/cgi-bin/amigo/go.cgi?view=details&depth=1&query=70374) |
|  |  | [positive regulation of I-kappaB kinase/NF-kappaB cascade](http://amigo.geneontology.org/cgi-bin/amigo/go.cgi?view=details&depth=1&query=43123) |
|  |  | [positive regulation of JNK cascade](http://amigo.geneontology.org/cgi-bin/amigo/go.cgi?view=details&depth=1&query=46330) |
|  |  | [positive regulation of NF-kappaB import into nucleus](http://amigo.geneontology.org/cgi-bin/amigo/go.cgi?view=details&depth=1&query=42346) |
|  |  | [positive regulation of Ras GTPase activity](http://amigo.geneontology.org/cgi-bin/amigo/go.cgi?view=details&depth=1&query=32320) |
|  |  | [positive regulation of T cell proliferation](http://amigo.geneontology.org/cgi-bin/amigo/go.cgi?view=details&depth=1&query=42102) |
|  |  | [positive regulation of T-helper 1 cell differentiation](http://amigo.geneontology.org/cgi-bin/amigo/go.cgi?view=details&depth=1&query=45627) |
|  |  | [positive regulation of cell motility](http://amigo.geneontology.org/cgi-bin/amigo/go.cgi?view=details&depth=1&query=2000147) |
|  |  | [positive regulation of chemotaxis](http://amigo.geneontology.org/cgi-bin/amigo/go.cgi?view=details&depth=1&query=50921) |
|  |  | [positive regulation of dendritic cell antigen processing and presentation](http://amigo.geneontology.org/cgi-bin/amigo/go.cgi?view=details&depth=1&query=2606) |
| *CD14* | CD14 molecule |  |
|  |  | [I-kappaB kinase/NF-kappaB cascade](http://amigo.geneontology.org/cgi-bin/amigo/go.cgi?view=details&depth=1&query=7249) |
|  |  | [MyD88-dependent toll-like receptor signaling pathway](http://amigo.geneontology.org/cgi-bin/amigo/go.cgi?view=details&depth=1&query=2755) |
|  |  | [MyD88-independent toll-like receptor signaling pathway](http://amigo.geneontology.org/cgi-bin/amigo/go.cgi?view=details&depth=1&query=2756) |
|  |  | [TRIF-dependent toll-like receptor signaling pathway](http://amigo.geneontology.org/cgi-bin/amigo/go.cgi?view=details&depth=1&query=35666) |
|  |  | [apoptotic process](http://amigo.geneontology.org/cgi-bin/amigo/go.cgi?view=details&depth=1&query=6915) |
|  |  | [cell surface receptor signaling pathway](http://amigo.geneontology.org/cgi-bin/amigo/go.cgi?view=details&depth=1&query=7166) |
|  |  | [cellular response to lipopolysaccharide](http://amigo.geneontology.org/cgi-bin/amigo/go.cgi?view=details&depth=1&query=71222) |
|  |  | [cellular response to lipoteichoic acid](http://amigo.geneontology.org/cgi-bin/amigo/go.cgi?view=details&depth=1&query=71223) |
|  |  | [inflammatory response](http://amigo.geneontology.org/cgi-bin/amigo/go.cgi?view=details&depth=1&query=6954) |
|  |  | [innate immune response](http://amigo.geneontology.org/cgi-bin/amigo/go.cgi?view=details&depth=1&query=45087) |
|  |  | [phagocytosis](http://amigo.geneontology.org/cgi-bin/amigo/go.cgi?view=details&depth=1&query=6909) |
|  |  | [positive regulation of cytokine secretion](http://amigo.geneontology.org/cgi-bin/amigo/go.cgi?view=details&depth=1&query=50715) |
|  |  | [positive regulation of endocytosis](http://amigo.geneontology.org/cgi-bin/amigo/go.cgi?view=details&depth=1&query=45807) |
|  |  | [positive regulation of tumor necrosis factor production](http://amigo.geneontology.org/cgi-bin/amigo/go.cgi?view=details&depth=1&query=32760) |
|  |  | [response to heat](http://amigo.geneontology.org/cgi-bin/amigo/go.cgi?view=details&depth=1&query=9408) |
|  |  | [toll-like receptor 2 signaling pathway](http://amigo.geneontology.org/cgi-bin/amigo/go.cgi?view=details&depth=1&query=34134) |
|  |  | [toll-like receptor 3 signaling pathway](http://amigo.geneontology.org/cgi-bin/amigo/go.cgi?view=details&depth=1&query=34138) |
|  |  | [toll-like receptor 4 signaling pathway](http://amigo.geneontology.org/cgi-bin/amigo/go.cgi?view=details&depth=1&query=34142) |
|  |  | [toll-like receptor TLR1:TLR2 signaling pathway](http://amigo.geneontology.org/cgi-bin/amigo/go.cgi?view=details&depth=1&query=38123) |
|  |  | [toll-like receptor TLR6:TLR2 signaling pathway](http://amigo.geneontology.org/cgi-bin/amigo/go.cgi?view=details&depth=1&query=38124) |
|  |  | [toll-like receptor signaling pathway](http://amigo.geneontology.org/cgi-bin/amigo/go.cgi?view=details&depth=1&query=2224) |
| *CIB1* | Calcium and integrin binding 1 (calmyrin) |  |
|  |  | [apoptotic process](http://amigo.geneontology.org/cgi-bin/amigo/go.cgi?view=details&depth=1&query=6915) |
|  |  | [cell adhesion](http://amigo.geneontology.org/cgi-bin/amigo/go.cgi?view=details&depth=1&query=7155) |
|  |  | [cellular response to DNA damage stimulus](http://amigo.geneontology.org/cgi-bin/amigo/go.cgi?view=details&depth=1&query=6974) |
|  |  | [double-strand break repair](http://amigo.geneontology.org/cgi-bin/amigo/go.cgi?view=details&depth=1&query=6302) |
|  |  | [endomitotic cell cycle](http://amigo.geneontology.org/cgi-bin/amigo/go.cgi?view=details&depth=1&query=7113) |
|  |  | [extrinsic apoptotic signaling pathway](http://amigo.geneontology.org/cgi-bin/amigo/go.cgi?view=details&depth=1&query=97191) |
|  |  | [negative regulation of cell proliferation](http://amigo.geneontology.org/cgi-bin/amigo/go.cgi?view=details&depth=1&query=8285) |
|  |  | [positive regulation of calcineurin-NFAT signaling cascade](http://amigo.geneontology.org/cgi-bin/amigo/go.cgi?view=details&depth=1&query=70886) |
|  |  | [positive regulation of establishment of protein localization to plasma membrane](http://amigo.geneontology.org/cgi-bin/amigo/go.cgi?view=details&depth=1&query=90004) |
| *DNAJA1* | DnaJ (Hsp40) homolog, subfamily A, member 1 |  |
|  |  | [androgen receptor signaling pathway](http://amigo.geneontology.org/cgi-bin/amigo/go.cgi?view=details&depth=1&query=30521) |
|  |  | [protein folding](http://amigo.geneontology.org/cgi-bin/amigo/go.cgi?view=details&depth=1&query=6457) |
|  |  | [response to heat](http://amigo.geneontology.org/cgi-bin/amigo/go.cgi?view=details&depth=1&query=9408) |
|  |  | [response to unfolded protein](http://amigo.geneontology.org/cgi-bin/amigo/go.cgi?view=details&depth=1&query=6986) |
|  |  | [sperm motility](http://amigo.geneontology.org/cgi-bin/amigo/go.cgi?view=details&depth=1&query=30317) |
|  |  | [spermatogenesis](http://amigo.geneontology.org/cgi-bin/amigo/go.cgi?view=details&depth=1&query=7283) |
| *DSG1* | Desmoglein 1 |  |
|  |  | [apoptotic process](http://amigo.geneontology.org/cgi-bin/amigo/go.cgi?view=details&depth=1&query=6915) |
|  |  | [calcium-dependent cell-cell adhesion](http://amigo.geneontology.org/cgi-bin/amigo/go.cgi?view=details&depth=1&query=16339) |
|  |  | [cell-cell adhesion](http://amigo.geneontology.org/cgi-bin/amigo/go.cgi?view=details&depth=1&query=16337) |
|  |  | [cell-cell junction assembly](http://amigo.geneontology.org/cgi-bin/amigo/go.cgi?view=details&depth=1&query=7043) |
|  |  | [cellular component disassembly involved in execution phase of apoptosis](http://amigo.geneontology.org/cgi-bin/amigo/go.cgi?view=details&depth=1&query=6921) |
|  |  | [homophilic cell adhesion](http://amigo.geneontology.org/cgi-bin/amigo/go.cgi?view=details&depth=1&query=7156) |
|  |  | [maternal process involved in female pregnancy](http://amigo.geneontology.org/cgi-bin/amigo/go.cgi?view=details&depth=1&query=60135) |
|  |  | [protein stabilization](http://amigo.geneontology.org/cgi-bin/amigo/go.cgi?view=details&depth=1&query=50821) |
|  |  | [response to progesterone stimulus](http://amigo.geneontology.org/cgi-bin/amigo/go.cgi?view=details&depth=1&query=32570) |
| *ERRFI1* | ERBB receptor feedback inhibitor 1 |  |
|  |  | [lung alveolus development](http://amigo.geneontology.org/cgi-bin/amigo/go.cgi?view=details&depth=1&query=48286) |
|  |  | [lung epithelium development](http://amigo.geneontology.org/cgi-bin/amigo/go.cgi?view=details&depth=1&query=60428) |
|  |  | [lung vasculature development](http://amigo.geneontology.org/cgi-bin/amigo/go.cgi?view=details&depth=1&query=60426) |
|  |  | [negative regulation of epidermal growth factor-activated receptor activity](http://amigo.geneontology.org/cgi-bin/amigo/go.cgi?view=details&depth=1&query=7175) |
|  |  | [negative regulation of epidermal growth factor-activated receptor activity](http://amigo.geneontology.org/cgi-bin/amigo/go.cgi?view=details&depth=1&query=7175) |
|  |  | [negative regulation of protein autophosphorylation](http://amigo.geneontology.org/cgi-bin/amigo/go.cgi?view=details&depth=1&query=31953) |
|  |  | [positive regulation of Rho GTPase activity](http://amigo.geneontology.org/cgi-bin/amigo/go.cgi?view=details&depth=1&query=32321) |
|  |  | [regulation of keratinocyte differentiation](http://amigo.geneontology.org/cgi-bin/amigo/go.cgi?view=details&depth=1&query=45616) |
|  |  | [response to stress](http://amigo.geneontology.org/cgi-bin/amigo/go.cgi?view=details&depth=1&query=6950) |
|  |  | [skin morphogenesis](http://amigo.geneontology.org/cgi-bin/amigo/go.cgi?view=details&depth=1&query=43589) |
| *FADS2* | Fatty acid desaturase 2 |  |
|  |  | [alpha-linolenic acid metabolic process](http://amigo.geneontology.org/cgi-bin/amigo/go.cgi?view=details&depth=1&query=36109) |
|  |  | [linoleic acid metabolic process](http://amigo.geneontology.org/cgi-bin/amigo/go.cgi?view=details&depth=1&query=43651) |
|  |  | [small molecule metabolic process](http://amigo.geneontology.org/cgi-bin/amigo/go.cgi?view=details&depth=1&query=44281) |
|  |  | [unsaturated fatty acid biosynthetic process](http://amigo.geneontology.org/cgi-bin/amigo/go.cgi?view=details&depth=1&query=6636) |
|  |  | [unsaturated fatty acid metabolic process](http://amigo.geneontology.org/cgi-bin/amigo/go.cgi?view=details&depth=1&query=33559) |
| *FOXA3* | Forkhead box A3 |  |
|  |  | [cellular glucose homeostasis](http://amigo.geneontology.org/cgi-bin/amigo/go.cgi?view=details&depth=1&query=1678) |
|  |  | [cellular response to starvation](http://amigo.geneontology.org/cgi-bin/amigo/go.cgi?view=details&depth=1&query=9267) |
|  |  | [chromatin modification](http://amigo.geneontology.org/cgi-bin/amigo/go.cgi?view=details&depth=1&query=16568) |
|  |  | [embryo development](http://amigo.geneontology.org/cgi-bin/amigo/go.cgi?view=details&depth=1&query=9790) |
|  |  | [endocrine pancreas development](http://amigo.geneontology.org/cgi-bin/amigo/go.cgi?view=details&depth=1&query=31018) |
|  |  | [negative regulation of transcription from RNA polymerase II promoter](http://amigo.geneontology.org/cgi-bin/amigo/go.cgi?view=details&depth=1&query=122) |
|  |  | [neuron fate specification](http://amigo.geneontology.org/cgi-bin/amigo/go.cgi?view=details&depth=1&query=48665) |
|  |  | [pattern specification process](http://amigo.geneontology.org/cgi-bin/amigo/go.cgi?view=details&depth=1&query=7389) |
|  |  | [positive regulation of neuron differentiation](http://amigo.geneontology.org/cgi-bin/amigo/go.cgi?view=details&depth=1&query=45666) |
|  |  | [positive regulation of transcription from RNA polymerase II promoter](http://amigo.geneontology.org/cgi-bin/amigo/go.cgi?view=details&depth=1&query=45944) |
|  |  | [regulation of hormone levels](http://amigo.geneontology.org/cgi-bin/amigo/go.cgi?view=details&depth=1&query=10817) |
|  |  | [regulation of sequence-specific DNA binding transcription factor activity](http://amigo.geneontology.org/cgi-bin/amigo/go.cgi?view=details&depth=1&query=51090) |
|  |  | [spermatogenesis](http://amigo.geneontology.org/cgi-bin/amigo/go.cgi?view=details&depth=1&query=7283) |
|  |  | [tissue development](http://amigo.geneontology.org/cgi-bin/amigo/go.cgi?view=details&depth=1&query=9888) |
| *FXR1* | Fragile X mental retardation, autosomal homolog 1 |  |
|  |  | [apoptotic process](http://amigo.geneontology.org/cgi-bin/amigo/go.cgi?view=details&depth=1&query=6915) |
|  |  | [cell differentiation](http://amigo.geneontology.org/cgi-bin/amigo/go.cgi?view=details&depth=1&query=30154) |
|  |  | [muscle organ development](http://amigo.geneontology.org/cgi-bin/amigo/go.cgi?view=details&depth=1&query=7517) |
|  |  | negative regulation of translation |
| *GKAP1* | G kinase anchoring protein 1 |  |
|  |  | signal transduction |
| *GNS* | Glucosamine (N-acetyl)-6-sulfatase |  |
|  |  | [carbohydrate metabolic process](http://amigo.geneontology.org/cgi-bin/amigo/go.cgi?view=details&depth=1&query=5975) |
|  |  | [glycosaminoglycan catabolic process](http://amigo.geneontology.org/cgi-bin/amigo/go.cgi?view=details&depth=1&query=6027) |
|  |  | [glycosaminoglycan metabolic process](http://amigo.geneontology.org/cgi-bin/amigo/go.cgi?view=details&depth=1&query=30203) |
|  |  | [keratan sulfate catabolic process](http://amigo.geneontology.org/cgi-bin/amigo/go.cgi?view=details&depth=1&query=42340) |
|  |  | [keratan sulfate metabolic process](http://amigo.geneontology.org/cgi-bin/amigo/go.cgi?view=details&depth=1&query=42339) |
|  |  | [small molecule metabolic process](http://amigo.geneontology.org/cgi-bin/amigo/go.cgi?view=details&depth=1&query=44281) |
| *GPBP1* | GC-rich promoter binding protein 1 |  |
|  |  | [positive regulation of transcription, DNA-dependent](http://amigo.geneontology.org/cgi-bin/amigo/go.cgi?view=details&depth=1&query=45893) |
|  |  | [transcription, DNA-dependent](http://amigo.geneontology.org/cgi-bin/amigo/go.cgi?view=details&depth=1&query=6351) |
| *HEYL* | Hairy/enhancer-of-split related with YRPW motif-like |  |
|  |  | [Notch signaling pathway](http://amigo.geneontology.org/cgi-bin/amigo/go.cgi?view=details&depth=1&query=7219) |
|  |  | [atrioventricular valve morphogenesis](http://amigo.geneontology.org/cgi-bin/amigo/go.cgi?view=details&depth=1&query=3181) |
|  |  | [cardiac epithelial to mesenchymal transition](http://amigo.geneontology.org/cgi-bin/amigo/go.cgi?view=details&depth=1&query=60317) |
|  |  | [cardiac ventricle morphogenesis](http://amigo.geneontology.org/cgi-bin/amigo/go.cgi?view=details&depth=1&query=3208) |
|  |  | [cellular response to BMP stimulus](http://amigo.geneontology.org/cgi-bin/amigo/go.cgi?view=details&depth=1&query=71773) |
|  |  | [endocardial cushion morphogenesis](http://amigo.geneontology.org/cgi-bin/amigo/go.cgi?view=details&depth=1&query=3203) |
|  |  | epithelial to mesenchymal transition involved in endocardial cushion formation |
|  |  | [glomerulus development](http://amigo.geneontology.org/cgi-bin/amigo/go.cgi?view=details&depth=1&query=32835) |
|  |  | [mesenchymal cell development](http://amigo.geneontology.org/cgi-bin/amigo/go.cgi?view=details&depth=1&query=14031) |
|  |  | [negative regulation of androgen receptor activity](http://amigo.geneontology.org/cgi-bin/amigo/go.cgi?view=details&depth=1&query=2000824) |
|  |  | [negative regulation of androgen receptor signaling pathway](http://amigo.geneontology.org/cgi-bin/amigo/go.cgi?view=details&depth=1&query=60766) |
|  |  | [negative regulation of transcription, DNA-dependent](http://amigo.geneontology.org/cgi-bin/amigo/go.cgi?view=details&depth=1&query=45892) |
|  |  | [outflow tract morphogenesis](http://amigo.geneontology.org/cgi-bin/amigo/go.cgi?view=details&depth=1&query=3151) |
|  |  | [positive regulation of neuron differentiation](http://amigo.geneontology.org/cgi-bin/amigo/go.cgi?view=details&depth=1&query=45666) |
|  |  | [positive regulation of transcription from RNA polymerase II promoter](http://amigo.geneontology.org/cgi-bin/amigo/go.cgi?view=details&depth=1&query=45944) |
|  |  | proximal tubule development |
|  |  | [pulmonary valve morphogenesis](http://amigo.geneontology.org/cgi-bin/amigo/go.cgi?view=details&depth=1&query=3184) |
|  |  | [skeletal muscle cell differentiation](http://amigo.geneontology.org/cgi-bin/amigo/go.cgi?view=details&depth=1&query=35914) |
|  |  | [ventricular septum morphogenesis](http://amigo.geneontology.org/cgi-bin/amigo/go.cgi?view=details&depth=1&query=60412) |
| *IP6K2* | Inositol hexakisphosphate kinase 2 |  |
|  |  | [cytokine-mediated signaling pathway](http://amigo.geneontology.org/cgi-bin/amigo/go.cgi?view=details&depth=1&query=19221) |
|  |  | [inositol phosphate metabolic process](http://amigo.geneontology.org/cgi-bin/amigo/go.cgi?view=details&depth=1&query=43647) |
|  |  | [negative regulation of cell growth](http://amigo.geneontology.org/cgi-bin/amigo/go.cgi?view=details&depth=1&query=30308) |
|  |  | [phosphate ion transport](http://amigo.geneontology.org/cgi-bin/amigo/go.cgi?view=details&depth=1&query=6817) |
|  |  | [phosphatidylinositol phosphorylation](http://amigo.geneontology.org/cgi-bin/amigo/go.cgi?view=details&depth=1&query=46854) |
|  |  | [positive regulation of apoptotic process](http://amigo.geneontology.org/cgi-bin/amigo/go.cgi?view=details&depth=1&query=43065) |
|  |  | [small molecule metabolic process](http://amigo.geneontology.org/cgi-bin/amigo/go.cgi?view=details&depth=1&query=44281) |
|  |  | [type I interferon-mediated signaling pathway](http://amigo.geneontology.org/cgi-bin/amigo/go.cgi?view=details&depth=1&query=60337) |

**Supplementary Table 2.** FAME GO biological processes associated with up-regulated miRNAs under SNEB

| **miRNA** | **GO term** | **p-value** | **q-value** | **z-score** | **enrichment factor** | **Targets** | **HG p value** |
| --- | --- | --- | --- | --- | --- | --- | --- |
| mir-17-5p |  |  |  |  |  |  |  |
|  | regulation of cell cycle | 0.0001 | 0.0648 | 4 | 1.63 | 42 | 0.000682 |
|  | cell cycle arrest | 0.0002 | 0.0486 | 4.03 | 2.34 | 15 | 0.005124 |
|  | negative regulation of progression through cell cycle | 0.0002 | 0.0324 | 3.77 | 1.73 | 31 | 0.000604 |
|  | ER-nuclear signaling pathway | 0.0003 | 0.0405 | 4.01 | 3.58 | 5 | 0.061275 |
|  | protein kinase cascade | 0.001 | 0.081 | 3.23 | 1.44 | 49 | 0.000432 |
|  | unfolded protein response | 0.0012 | 0.09 | 3.68 | 3.93 | 3 | 0.148206 |
|  | intracellular signaling cascade | 0.0014 | 0.094 | 3.14 | 1.25 | 124 | 0.000101 |
|  | regulation of GTPase activity | 0.0026 | 0.1271 | 3.06 | 1.93 | 15 | 0.003838 |
|  | sterol metabolism | 0.0027 | 0.125 | 3.2 | 2.33 | 9 | 0.052088 |
|  | cholesterol metabolism | 0.0029 | 0.1174 | 3.1 | 2.35 | 8 | 0.066757 |
|  | phosphate metabolism | 0.003 | 0.1124 | 2.71 | 1.24 | 91 | 9.16E-05 |
|  | positive regulation of cellular metabolism | 0.0041 | 0.1476 | 2.69 | 1.28 | 64 | 8.85E-05 |
|  | positive regulation of metabolism | 0.0052 | 0.162 | 2.62 | 1.27 | 67 | 0.000112 |
|  | di-, tri-valent inorganic cation homeostasis | 0.0054 | 0.1576 | 2.77 | 1.99 | 10 | 0.3262 |
|  | regulation of small GTPase mediated signal transduction | 0.0054 | 0.1507 | 2.77 | 1.57 | 28 | 2.72E-05 |
|  | transcription | 0.0054 | 0.1458 | 2.55 | 1.13 | 214 | 3.94E-07 |
|  | negative regulation of phosphate metabolism | 0.0061 | 0.1581 | 2.97 | 3.07 | 3 | 0.202438 |
|  | cation homeostasis | 0.0071 | 0.1632 | 2.72 | 1.87 | 12 | 0.265077 |
|  | regulation of transcription | 0.0075 | 0.1676 | 2.39 | 1.12 | 210 | 3.60E-07 |
|  | protein modification | 0.0076 | 0.1631 | 2.46 | 1.16 | 160 | 1.71E-05 |
|  | metal ion homeostasis | 0.0088 | 0.1728 | 2.58 | 1.85 | 11 | 0.274285 |
|  | regulation of cellular metabolism | 0.0089 | 0.1706 | 2.37 | 1.11 | 235 | 3.43E-07 |
|  | regulation of metabolism | 0.0096 | 0.1728 | 2.36 | 1.11 | 247 | 3.45E-07 |
|  | protein amino acid dephosphorylation | 0.0104 | 0.1736 | 2.4 | 1.58 | 19 | 0.003511 |
|  | positive regulation of biosynthesis | 0.0109 | 0.1758 | 2.52 | 2.02 | 9 | 0.126439 |
|  | homeostasis | 0.0143 | 0.2163 | 2.22 | 1.42 | 26 | 0.268694 |
|  | phosphorylation | 0.015 | 0.2167 | 2.23 | 1.22 | 73 | 0.000764 |
|  | regulation of phosphorylation | 0.0153 | 0.2148 | 2.32 | 1.71 | 11 | 0.094397 |
|  | regulation of proteolysis | 0.0154 | 0.2123 | 2.61 | 3.12 | 3 | 0.078464 |
|  | cell ion homeostasis | 0.0155 | 0.2099 | 2.3 | 1.69 | 12 | 0.341117 |
|  | glucose homeostasis | 0.0159 | 0.2109 | 2.5 | 2.56 | 5 | 0.033488 |
|  | protein amino acid phosphorylation | 0.0178 | 0.2255 | 2.14 | 1.22 | 68 | 0.000219 |
|  | ion homeostasis | 0.0186 | 0.2318 | 2.21 | 1.64 | 13 | 0.328259 |
|  | regulation of cell growth | 0.0187 | 0.228 | 2.19 | 1.54 | 19 | 0.020886 |
|  | glycosphingolipid metabolism | 0.0199 | 0.2339 | 2.4 | 2.81 | 3 | 0.123098 |
|  | small GTPase mediated signal transduction | 0.0227 | 0.2536 | 2.03 | 1.28 | 48 | 0.002701 |
|  | signal transduction | 0.0229 | 0.2479 | 1.98 | 1.1 | 226 | 0.001202 |
|  | regulation of hormone secretion | 0.0313 | 0.2935 | 2.09 | 2.27 | 4 | 0.094266 |
|  | positive regulation of amino acid metabolism | 0.0325 | 0.3013 | 2 | 1.87 | 6 | 0.075973 |
|  | enzyme linked receptor protein signaling pathway | 0.0336 | 0.3071 | 1.87 | 1.24 | 46 | 0.00058 |
|  | regulation of phosphate metabolism | 0.0365 | 0.3196 | 1.87 | 1.54 | 11 | 0.147173 |
|  | positive regulation of protein metabolism | 0.0386 | 0.3244 | 1.86 | 1.6 | 10 | 0.148064 |
|  | cholesterol homeostasis | 0.0451 | 0.3438 | 1.91 | 2.27 | 3 | 0.17482 |
|  | calcium ion homeostasis | 0.0472 | 0.3556 | 1.8 | 1.71 | 7 | 0.494794 |
|  | negative regulation of physiological process | 0.0479 | 0.3564 | 1.68 | 1.13 | 104 | 0.007844 |
|  | regulation of ion transport | 0.0485 | 0.3492 | 1.91 | 2.4 | 4 | 0.192943 |
| miR-31 |  |  |  |  |  |  |  |
|  | amino acid transport | 0.0011 | 0.0734 | 4.34 | 5.4 | 4 | 0.022233 |
|  | carboxylic acid transport | 0.0012 | 0.0641 | 4.03 | 4.69 | 5 | 0.010893 |
|  | cell growth | 0.0098 | 0.124 | 2.64 | 2.37 | 8 | 0.020023 |
|  | response to stress | 0.0231 | 0.2127 | 2.11 | 1.55 | 18 | 0.173549 |
|  | regulation of cell growth | 0.037 | 0.2823 | 2 | 2.13 | 6 | 0.055108 |
|  | negative regulation of growth | 0.0448 | 0.3236 | 1.98 | 2.49 | 4 | 0.040537 |
|  | growth | 0.0463 | 0.325 | 1.8 | 1.7 | 9 | 0.093659 |
| mir-140 |  |  |  |  |  |  |  |
|  | positive regulation of cell proliferation | 0.0037 | 0.5681 | 3.21 | 2.2 | 14 | 0.000876 |
|  | regulation of cell proliferation | 0.0045 | 0.4545 | 2.85 | 1.73 | 22 | 0.000871 |
|  | response to nutrient levels | 0.0449 | 0.3887 | 2.05 | 2.95 | 3 | 0.071954 |
|  | activation of protein kinase activity | 0.0452 | 0.38 | 2.01 | 2.9 | 3 | 0.1387 |
